# The core microbiome of sessile ciliate *Stentor coeruleus* is not shaped by the environment

**DOI:** 10.1038/s41598-019-47701-8

**Published:** 2019-08-06

**Authors:** Olivia Lanzoni, Andrey Plotnikov, Yuri Khlopko, Giulio Munz, Giulio Petroni, Alexey Potekhin

**Affiliations:** 1 Department of Biology, University of Pisa, Pisa, Italy; 2 Center of Shared Scientific Equipment, Institute for Cellular and Intracellular Symbiosis, Ural Division of RAS, Orenburg, Russia; 3 Department of Civil and Environmental Engineering, University of Florence, Florence, Italy; 4 Faculty of Biology, Saint Petersburg State University, Saint Petersburg, Russia

**Keywords:** Microbial ecology, Microbial ecology, Microbiome, Water microbiology, Metagenomics

## Abstract

Microbiomes of multicellular organisms are one of the hottest topics in microbiology and physiology, while only few studies addressed bacterial communities associated with protists. Protists are widespread in all environments and can be colonized by plethora of different bacteria, including also human pathogens. The aim of this study was to characterize the prokaryotic community associated with the sessile ciliate *Stentor coeruleus*. 16S rRNA gene metabarcoding was performed on single cells of *S*. *coeruleus* and on their environment, water from the sewage stream. Our results showed that the prokaryotic community composition differed significantly between *Stentor* cells and their environment. The core microbiome common for all ciliate specimens analyzed could be defined, and it was composed mainly by representatives of bacterial genera which include also potential human pathogens and commensals, such as *Neisseria*, *Streptococcus*, *Capnocytophaga*, *Porphyromonas*. Numerous 16S rRNA gene contigs belonged to endosymbiont “*Candidatus* Megaira polyxenophila”. Our data suggest that each ciliate cell can be considered as an ecological microniche harboring diverse prokaryotic organisms. Possible benefits for persistence and transmission in nature for bacteria associated with protists are discussed. Our results support the hypothesis that ciliates attract potentially pathogenic bacteria and play the role of natural reservoirs for them.

## Introduction

All possible forms of coexistence of prokaryotes with metazoan organisms became one of the most rapidly developing research fields in microbiology, and also in physiology^1,2^. Protists may also host associated bacteria, but their microbiomes still are not investigated, and ciliates seem to be perfect candidates for this purpose. They are relatively big and bacterivorous, a number of representatives of this abundant phylum are easily recognizable and can be maintained in cultures. Ciliates have been intensively studied in wastewaters, focusing on their role as indicators of process efficiency^3–5^ and as detectors of heavy metal pollution^6,7^. Indeed, ciliates as filter-feeders are also efficient removers of some pathogens^8–11^, and contribute, together with disinfection systems, in pulling down the microbial load^12,13^, thus improving the quality of the effluent discharged by wastewater treatment plants^14^. At the same time, bacteria may benefit in finding protection inside the host cell from disinfection systems and chemical substances used to reduce bacterial load^12,13,15^. The phagotrophic activity of ciliates allows natural entrance of bacteria into the eukaryotic host, thus enabling the establishment of symbiotic associations with bacteria, also occasionally pathogenic ones^16^. Indeed, the ciliate cell offers a great variety of intracellular compartments suitable for bacterial colonization, and symbiotic bacteria have been described in the ciliates’ cytoplasm, nuclear apparatus, mitochondria and even in perinuclear space^17^.

Ciliates often host endosymbionts phylogenetically related to pathogenic bacteria belonging to the families *Rickettsiaceae*^18–20^ or *Francisellaceae*^21,22^. Symbiotic associations can be classified according to the stability of bacteria and ciliate interaction in three categories: permanent, highly infectious, and accidental^17^. The first two groups comprise all associations, which were recorded more than once in literature, and have been studied from ecological and evolutionary points of view. However, accidental invaders are never considered as true symbionts, such bacteria are likely poorly adapted for symbiotic persistence, and cannot be maintained for a long time within the host cell^17^. Such invaders usually have been reported just once^17^, and the study of these temporary occasional associations with “classical” microbiological and molecular techniques is rather difficult, as the associations are very unstable and promptly get lost. Several studies reported that in ciliates the establishment of symbiotic associations with pathogenic bacteria can be experimentally induced^23^. For example, ciliates under certain conditions may host *Legionella pneumophila*^24,25^, that sometimes leads to increase of virulence of these bacteria for human cells^26^. Internalized *Listeria* remain infectious in cysts of *Tetrahymena*^27^, and pathogenic *Escherichia coli* survives after passage through *Tetrahymena* cells^28^. However, such systems do not appear to be stable. The role of protists as environmental reservoirs of pathogenic bacteria has been elucidated more in amoebae, in which several pathogens have been found^29,30^, for example *Francisella tularensis*^31,32^, *Vibrio cholerae*^33^, *E*. *coli*^29^, *Chlamydia*^34^, *Mycobacterium*^35–37^, and *Listeria monocytogenes*^38^. Thus, in some cases, pathogenic bacteria also are a part of prokaryotic communities associated to protists, but for most of them the mode of maintenance and propagation in environment remains unclear.

However, contribution of bacteria in survival and environmental adaptation of protists, as well as the role of protists as reservoirs for the pools of microorganisms remains poorly understood. The studies of protistan microbiomes just start to be addressed with the diffusion of new technologies, such as Next Generation Sequencing (NGS). The characterization of the bacterial consortium associated with the Antarctic marine ciliate *Euplotes focardii* has been reported^39^. It was shown that even during long maintenance of ciliates in laboratory conditions they still keep a rather variegated set of initial bacterial cohabitants. Recently, Illumina sequencing was applied to discriminate between bacteria of the rumen fluid and symbiotic prokaryotes of ruminant ciliates^40^. The results of the pilot study showing that marine and freshwater ciliates harbour distinct microbial communities were reported in the latest publication^41^, and such prokaryotic communities were analyzed for several *Paramecium* samples^42^. However, up to now just two latter works remain the only studies of prokaryotic communities associated with single ciliate cells by NGS. This gap in knowledge extends also to other groups of protists, as there were only few attempts to assess the microbiomes of free-living amoebae^43,44^ applying NGS, and in several works bacterial diversity in association with protists was estimated by cloning and sequencing^45–49^. Also, few reports analyzed microbial consortia associated to cyanobacteria^50–52^ proving that even bacteria may organize and maintain stable communities of cohabiting prokaryotes. Nevertheless, all these few studies assume that unicellular organisms do have their own microbiomes.

**Figure 1:**
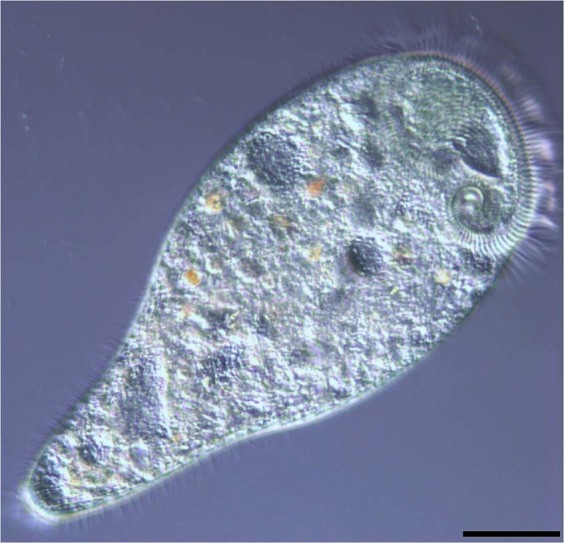
*In vivo* morphology of *Stentor coeruleus*. Nikon Eclipse Ni, DIC microscopy. Scale bar = 50 µm.

Herein, we investigated the microbiomes of the sessile ciliates *Stentor coeruleus* (Fig. 1), isolated from a sewage stream, applying 16S rRNA gene metabarcoding approach on single ciliate cells. We propose to apply the term “core microbiome” for ciliates. Core microbiomes are defined as the “assemblages of microorganisms, active or inactive, associated with a certain habitat”^53^. Other authors suppose that core microbiome is the interacting subset of the total microbiome which fulfill a certain active function^54^. We suppose that bacteria composing core microbiome of a ciliate are prone to interact with eukaryotic cells. We also provide further support to the hypothesis that ciliates may host some opportunistic bacteria and should be considered as potential reservoirs of human pathogens.

## Results

A total number of 554260 reads were assembled, and after denoising and chimera filtering the obtained contigs clustered at 97% similarity threshold in 473 OTUs (for further information see Supplementary Table 1).

**Figure 2:**
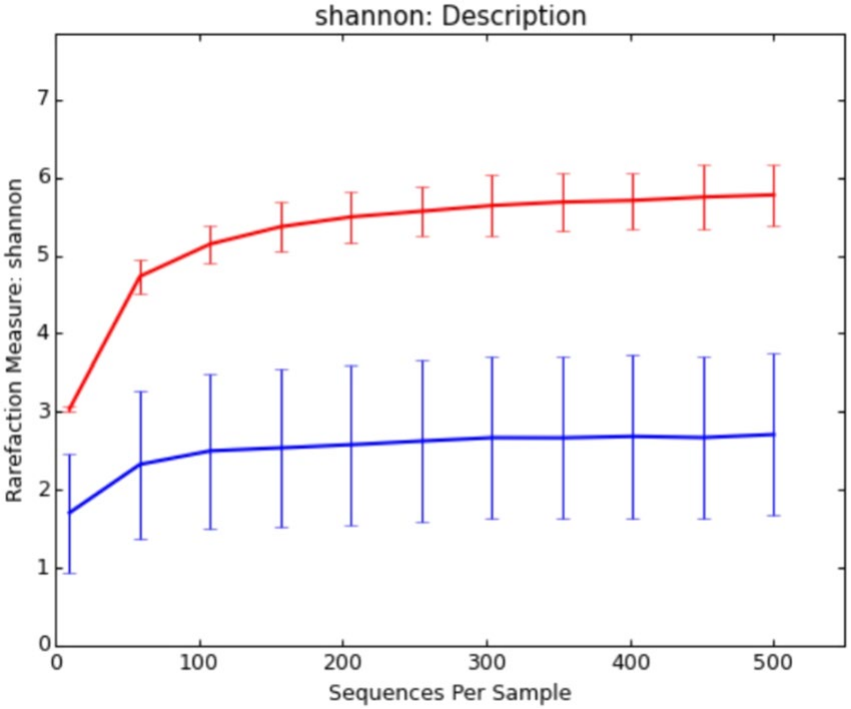
Rarefaction analysis of the studied samples. The average values of Shannon index are reported for the environmental samples (red) and for the *Stentor* cells (blue). The curves were generated basing on a 97% threshold level sequence similarity of OTUs.

Average Shannon diversity index was calculated to assess diversity of the bacterial communities from the environment and those associated with the *Stentor* cells (Fig. 2). The environmental prokaryotic community was more than twice as much diverse as total prokaryotic community associated with the ciliates: average value of the environmental community richness index was 5.26 ± 0.30, while for the stentors it was 2.51 ± 0.99. Moreover, both rarefaction curves reached plateau indicating that OTUs forming respective communities were almost completely determined.

**Figure 3:**
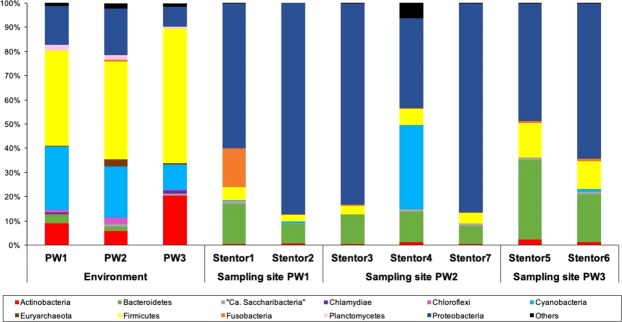
Composition of the prokaryotic communities from the environments and of *Stentor coeruleus* cells microbiomes. Relative abundances of the prokaryotic phyla are represented for each sample. Unclassified phyla are grouped in “Others”.

In total representatives of 11 phyla were found, and among them 8 were abundant in all environment samples, while 6 phyla were the most plentiful in *Stentor* cells (Fig. 3). Bacterial community composition differed between the environments and the *Stentor* cells. Indeed, *Firmicutes* was the most abundant phylum in the environments (45.1%, average percentage of contigs), followed by *Cyanobacteria* (19.3%), *Proteobacteria* (14.6%), *Actinobacteria* (11.7%), *Bacteroidetes* (1.9%), *Planctomycetes* (1.7%), *Chloroflexi* (1.3%), and *Euryarcheota* (1.2%). On the contrary, the most abundant phylum in *Stentor* cells was *Proteobacteria* (66.7%), followed by less copious *Bacteroidetes* (15.6%), *Firmicutes* (6.9%), *Cyanobacteria* (5.3%), *Fusobacteria* (2.7%), and *Actinobacteria* (1.0%).

All OTUs obtained and assigned to certain bacteria were categorized according to the ecological niche as belonging to free-living bacteria (i.e. all those bacteria which live in the environment and are not known to form any kind of symbiotic relations), human commensals (i.e. bacteria normally associated to healthy human individuals), potential pathogens or bacteria related to pathogens (i.e. those bacteria causing diseases in humans or animals), and symbionts (i.e. known as obligate intracellular bacteria) (Table 1). The set of OTUs in the environmental samples was composed of a number of free-living genera, which are ubiquitous and normally present in the environment, such as bacteria from the order *Rhizobiales* (uncultured MNG7), uncultured *Actinobacteria* (bacterium PeM15), uncultured *Cyanobacteria,* uncultured *Clostridiales*, and some *Firmicutes*. Some potential pathogens and opportunistic bacteria like *Streptococcus* sp. were also detected in the environmental samples. Only few OTUs found in the environments were rather frequent also in the ciliate samples, though showing different average percentages (Table 1). The OTU belonging to uncultured *Cyanobacteria*, (very prevalent in Stentor4 cell), was identified as the chloroplast sequence of the microalga *Mychonastes homosphaera* (*Chlorophyceae*), which once has been found in symbiotic association with *Stentor polymorphus*^55^. In the same *Stentor* cell, OTU belonging to *Holospora obtusa*, a specific bacterial endosymbiont of the ciliate *Paramecium caudatum*, was found, but its abundance was very low (0.20%) (for further information, see Supplementary Table 1).

**Table 1:** The most common OTUs retrieved from the environmental samples and from *Stentor* cell samples.

Ecological category	OTU Phylum	OTU Taxonomy	Environment			Sampling site PW1		Sampling site PW2			Sampling site PW3	
			PW1	PW2	PW3	Stentor 1	Stentor 2	Stentor 3	Stentor 4	Stentor 7	Stentor 5	Stentor 6
Free-living	*Actinobacteria*	uncultured bacterium PeM15	4.3%	1.8%	14.0%	0.0%	0.0%	0.0%	0.0%	0.0%	0.0%	0.0%
	*Cyanobacteria*	uncultured bacterium	24.0%	19.4%	10.5%	0.0%	0.6%	0.0%	32.9%	0.0%	0.0%	1.1%
	*Firmicutes*	uncultured *Clostridiales*	6.8%	6.0%	13.5%	0.0%	0.0%	0.0%	0.0%	0.0%	0.0%	0.0%
	*Proteobacteria*	uncultured bacterium MNG7	1.3%	2.6%	1.2%	0.3%	11.5%	0.0%	0.0%	0.1%	2.0%	11.4%
Human commensals	*Bacteroidetes*	*Porphyromonas* sp.	0.0%	0.0%	0.0%	2.0%	0.7%	1.7%	1.9%	0.9%	3.1%	4.7%
		*Capnocytophaga* sp.	0.0%	0.0%	0.0%	10.5%	4.6%	5.5%	7.9%	5.1%	27.3%	12.9%
	*Firmicutes*	uncultured bacterium *Christensenellaceae*	1.3%	6.3%	2.2%	0.0%	0.0%	0.0%	0.0%	0.0%	0.0%	0.0%
		*Faecalibacterium* sp.	0.1%	1.8%	5.4%	0.0%	0.0%	0.0%	0.0%	0.0%	0.0%	0.0%
		uncultured bacterium *Lachnospiraceae*	3.3%	1.6%	6.4%	0.0%	0.0%	0.0%	0.0%	0.0%	0.0%	0.0%
		*Subdoligranulum* sp.	7.9%	1.6%	3.0%	0.0%	0.1%	0.0%	0.0%	0.0%	0.0%	0.0%
	*Fusobacteria*	uncultured *Leptotrichiaceae*	0.0%	0.0%	0.0%	16.0%	0.0%	0.0%	0.0%	0.0%	0.0%	0.0%
Potential pathogens	*Firmicutes*	*Streptococcus* sp.	0.9%	0.9%	1.7%	3.5%	1.7%	2.6%	4.0%	3.0%	8.4%	8.0%
	*Proteobacteria*	*Neisseria* sp.	0.0%	0.0%	0.0%	1.4%	0.3%	1.1%	1.4%	2.1%	3.8%	4.9%
Specialized symbionts	*Proteobacteria*	“*Ca*. Megaira polyxenophila”	0.1%	6.6%	0.0%	55.8%	72.7%	79.7%	0.0%	82.2%	33.4%	39.6%

OTUs were classified down to genus level, when possible. Taxonomic affiliations and relative abundances in the environment and in association with *Stentor* cells are reported.

Numerous OTUs belonging to some uncultured representatives of *Neisseriaceae* and to the genus *Neisseria*, normally associated to human mucosa, were recorded in association with stentors, and never in the environmental controls. Interestingly, *Streptococcus* sp. appeared to be mostly associated to *Stentor* cells, despite its presence was detected also in the environment. The non-parametric Kruskal-Wallis test assessed that there was a significant difference in the distribution of OTUs between stentors and their environments (p < 0.05), thus showing that human commensals and potentially pathogenic bacteria were indeed preferentially associated to *Stentor* cells. However, the most abundant OTU in six of seven *Stentor* samples belonged to the widespread endosymbiotic bacterium “*Candidatus* Megaira polyxenophila” (*Alphaproteobacteria*, *Rickettsiales*), probably inhabiting *Stentor* cells (Table 1). OTUs belonging to this endosymbiont were also present in the environmental samples, likely due to the entrapment of some *Stentor* cells during water filtering.

**Figure 4:**
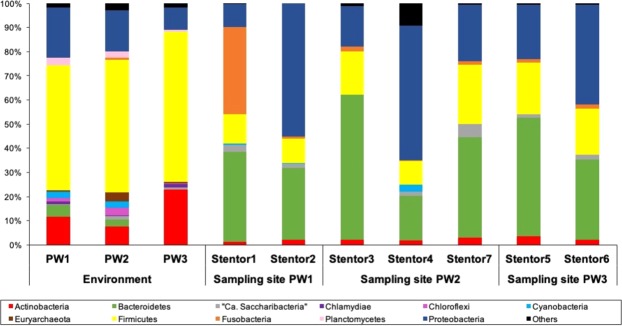
Composition of the prokaryotic communities from the environments and of *Stentor coeruleus* cells microbiomes after removal of presumable abundant symbionts. Relative abundances of the prokaryotic phyla are represented for each sample. Unclassified phyla are grouped in “Others”.

The microbial communities composition was reanalyzed after removing presumable symbionts OTUs, namely “*Ca*. Megaira polyxenophila” and the microalga chloroplast sequences, which reached 30–80% of the total number of reads in *Stentor* samples, thus, hiding the rest of bacterial diversity associated to ciliate cells. The total number of phyla did not change and corresponded to initial analysis, but their abundances shifted (Fig. 4). In the environment, *Firmucutes* (56.2%) remained the most abundant phylum, it was followed by *Proteobacteria* (15.8%), *Actinobacteria* (14.0%), *Bacteroidetes* (2.6%), *Planctomycetes* (2.2%), *Chloroflexi* (1.7%), and *Cyanobacteria* (1.7%). Removal of “*Ca*. Megaira polyxenophila” reads significantly reduced the fraction of *Proteobacteria* in the ciliates samples (32.0%), while the most abundant phyla associated with ciliates became *Bacteroidetes* (38.5%), then followed by *Firmicutes* (16.4%), *Fusobacteria* (6.3%), *Actinobacteria* (2.3%), and “*Ca*. Saccharibacteria” (2.1%), while *Cyanobacteria* (0.6%) became almost absent (Fig. 4).

After removing reads of the abundant symbionts, no new major taxa appeared in the environmental samples, while relative abundance of some OTUs increased significantly in the stentors samples (Table 2). The most common OTUs defined in the stentors samples belonged to the representatives of genera, which include human commensals or opportunistic pathogens.

**Table 2:** The most common OTUs retrieved from the environmental samples and from *Stentor* cell samples after removal of abundant symbionts’ OTUs.

Ecological category	OTU Phylum	OTU Taxonomy	Environment			Sampling site PW1		Sampling site PW2			Sampling site PW3	
			PW1	PW2	PW3	Stentor 1	Stentor 2	Stentor 3	Stentor 4	Stentor 7	Stentor 5	Stentor 6
Free-living	*Actinobacteria*	uncultured bacterium PeM15	5.7%	2.4%	15.7%	0.0%	0.0%	0.0%	0.0%	0.0%	0.0%	0.0%
	*Bacteroidetes*	uncultured bacterium *Chitinophagaceae*	0.2%	0.0%	0.0%	4.8%	7.3%	20.6%	0.1%	1.0%	0.4%	0.3%
	*“Ca*. Saccharibacteria”	uncultured bacterium “*Ca*. Saccharibacteria”	0.0%	1.4%	1.1%	1.2%	1.6%	0.0%	1.6%	1.7%	1.3%	2.1%
	*Firmicutes*	uncultured bacterium *Clostridiales*	9.0%	8.1%	15.1%	0.0%	0.0%	0.0%	0.0%	0.0%	0.0%	0.0%
	*Proteobacteria*	uncultured bacterium MNG7	1.8%	3.6%	1.3%	0.7%	42.9%	0.0%	0.0%	0.3%	3.1%	19.2%
		*Xanthobacter* sp.	0.0%	0.2%	0.0%	0.0%	0.0%	0.0%	18.2%	0.0%	0.0%	0.0%
Human commensals	*Actinobacteria*	*Rothia* sp.	0.0%	0.0%	0.0%	0.7%	2.1%	1.1%	0.8%	2.7%	3.1%	2.0%
	*Bacteroidetes*	*Bergeyella* sp.	0.0%	0.0%	0.0%	2.9%	1.0%	0.0%	0.8%	2.0%	1.4%	1.1%
		*Porphyromonas* sp.	0.0%	0.0%	0.0%	4.5%	2.6%	8.3%	2.8%	5.1%	4.6%	7.9%
		*Capnocytophaga* sp.	0.0%	0.0%	0.0%	23.7%	17.3%	27.2%	11.8%	28.7%	41.0%	21.8%
	*Firmicutes*	uncultured bacterium *Christensenellaceae*	1.8%	8.5%	2.5%	0.0%	0.0%	0.0%	0.0%	0.0%	0.0%	0.0%
		*Faecalibacterium* sp.	0.2%	2.4%	6.0%	0.0%	0.0%	0.0%	0.0%	0.0%	0.0%	0.0%
		uncultured bacterium *Lachnospiraceae*	4.3%	2.2%	7.2%	0.0%	0.0%	0.0%	0.0%	0.0%	0.2%	0.0%
		*Subdoligranulum* sp.	10.4%	2.2%	3.4%	0.0%	0.0%	0.0%	0.0%	0.0%	0.0%	0.0%
		*Veillonella* sp.	0.2%	0.0%	0.0%	3.8%	1.6%	2.2%	2.1%	4.4%	4.7%	3.2%
	*Fusobacteria*	uncultured *Leptotrichiaceae*	0.0%	0.0%	0.0%	36.1%	0.0%	0.0%	0.0%	0.0%	0.0%	0.0%
Potential pathogens	*Bacteroidetes*	*Prevotella* sp.	0.2%	0.0%	0.0%	1.4%	1.6%	2.8%	1.3%	4.1%	1.7%	1.0%
	*Firmicutes*	*Streptococcus* sp.	1.2%	1.2%	1.9%	7.9%	6.3%	12.8%	6.0%	16.9%	12.6%	13.4%
	*Proteobacteria*	*Lautropia* sp.	0.0%	0.0%	0.0%	1.2%	1.0%	4.4%	1.3%	1.4%	3.1%	1.6%
		*Neisseria* sp.	0.0%	0.0%	0.0%	3.1%	1.0%	5.6%	2.1%	11.8%	5.7%	8.2%
		uncultured bacterium *Neisseriaceae*	0.0%	0.0%	0.0%	2.6%	7.4%	3.3%	16.6%	5.1%	7.3%	7.0%
		*Haemophilus* sp.	0.0%	0.0%	0.0%	1.0%	1.6%	1.7%	0.5%	1.4%	0.7%	1.9%

OTUs were classified down to genus level, when possible. Taxonomic affiliations and relative abundances in the environment and in association with *Stentor* cells are reported.

Interestingly, a number of these bacteria were present, though in different percentages, in all seven or at least six *Stentor* samples (Table 2). We can assume that they form the core microbiome of *S*. *coeruleus* cells from the studied waterbody. This core microbiome included more than 10 bacterial genera, and just two of them, representatives of *Chitinophagaceae* and of “*Ca*. Saccharibacteria”, belong to the families considered as free-living. *Capnocytophaga* sp., *Streptococcus* sp., and representatives of *Neisseriaceae*, all known as opportunistic commensals of animals^56–60^, were predominant components of *Stentor* core microbiome. The ratios between members of core microbiome were different for all analyzed *Stentor* cells.

However, besides such widespread core microbiome components, some unique major bacteria were detected for some *Stentor* cells. For example, uncultured bacterium MNG7 was a major microbiome component of Stentor2 and Stentor6 specimens originating from two different sampling points. *Xanthobacter* sp. was the most abundant bacterium associated with Stentor4, and some bacteria from *Leptotrichiaceae* family were dominant in Stentor1 community. These bacteria, probably, can be considered as transitory components of *Stentor* microbiome.

**Figure 5:**
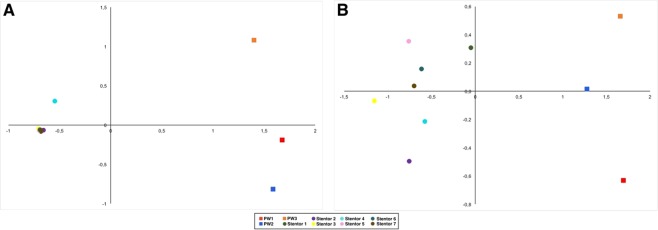
Results of Non-metric Multi-Dimensional Scaling based on the weighted Unifrac distance matrix. The original dataset (**A**), and the dataset after removal of the abundant symbionts (**B**).

In order to compare the prokaryotic community composition of the environments and ciliate cells, nmMDS was calculated on unweighted Unifrac distance matrix for all datasets (Fig. 5). The first dataset, comprising all symbiont contigs, showed a bright distinction between the environmental samples and the ciliate cells. Almost all stentors gathered together with the single exception of *Stentor*4, which located nearby other ciliates, but did not group with them, and was significantly distant from the environments (Fig. 5A). Indeed, this was the only *Stentor* specimen which had no “*Ca*. Megaira polyxenophila” endosymbionts, but contained symbiotic microalgae. After removal of symbionts OTUs, the environmental samples and the ciliates samples remained separated, and the ciliates still grouped together, though they became less congregated than before (Fig. 5B).

Several analyses were carried out to test if the prokaryotic community composition of the environments and of the *Stentor* cells was statistically different. ANOSIM test showed that prokaryotic community compositions were significantly different between the environmental samples and the *Stentor* samples in all datasets (p < 0.05), accordingly with the results of NMDS.

**Figure 6:**
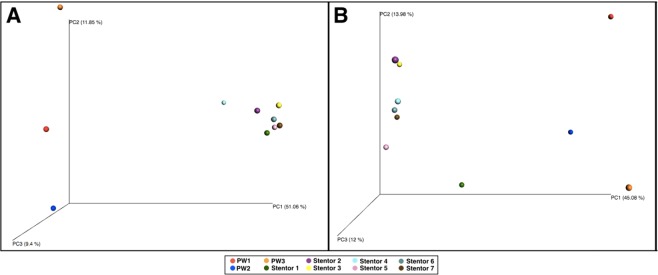
Results of Principal Component Analysis (PCoA). Percent of variation on the axis is indicated with PC. The original dataset (**A**), and the dataset after removal of the abundant symbionts (**B**).

PCoA was performed to determine whether prokaryotic communities of all samples were different among them, and across all datasets employed (Fig. 6). In the original dataset, all *Stentor* cells grouped together, and were separated from the environments (Fig. 6A). The other dataset, after removal of symbionts OTUs, showed that the ciliates microbiomes were in fact less homogeneous among themselves, but still reliably separated from environmental communities (Fig. 6B).

## Discussion

In this study, the microbiomes of single cells of free-living ciliate *Stentor coeruleus* were investigated using 16S rRNA gene metabarcoding. Few studies addressed this topic in the last years using different techniques. Cloning of 16S rRNA gene PCR products obtained from single ciliate cells^49,61^ allowed to disclose a complex microbial community, but this approach is prone to underestimate the real biodiversity of the sampled microbiome. The complete metagenome of the bacterial consortium associated with a massive culture of the Antarctic ciliate *Euplotes focardii* was analyzed by NGS, which showed the interactions between microbiome and the host cell^39^. Very recently, microbiomes of rumen^40^ and free-living^41,42^ ciliates were investigated using single-cell 16S rRNA gene metabarcoding. With our study, we provide another example of how NGS technologies can be applied on single cells of cultivable and uncultivable protists to study their microbiomes and to investigate the presence of potential symbionts.

Prokaryotic community disclosed in the environmental samples was diverse and especially rich with several bacterial phyla, first of all *Firmicutes*, *Actinobacteria* and *Proteobacteria*. Many bacteria known to be anaerobic (uncultivable *Clostridiales*), or previously found in activated sludge (uncultured bacteria from “*Ca*. Saccharibacteria”^62,63^) (Tables 1, 2) were detected. The presence of anaerobic microorganisms is probably due to relatively high volume of the bottom sludge which has been collected together with water in the samples. Although some ciliate cells inevitably remained on filters, and their bacteria were counted as “environmental”, bacterial communities associated with *Stentor* cells were totally different from those of the environments. The only major OTUs in common between environment and stentors were the protist endosymbiont “*Ca*. Megaira polyxenophila”, *Streptococcus sp*. (significantly more numerous in stentors), and uncultured *Rhizobiales* MNG7, probably inhabiting the stream. Majority of environmental bacteria were absent or negligible in the communities associated with *Stentor* cells.

Different *S*. *coeruleus* cells isolated from the neighboring locations of the sewage stream were characterized by rather diverse associated bacterial communities, which included several dozens of different representatives (Supplementary Table 1). The composition of ciliates’ bacterial community looked apparently very similar at a first glance (Fig. 3, Table 1). Indeed, in six *Stentor* cells of seven, the presence of the bacterial endosymbiont “*Ca*. Megaira polyxenophila” was detected (Table 1). This obligatory intracellular bacterium was described in several unicellular organisms, mostly ciliates, and is phylogenetically closely related to the pathogen *Rickettsia*^64^. Unfortunately, we were not able to confirm directly “*Ca*. Megaira polyxenophila” presence inside the studied *Stentor* cells, as at the moment of OTUs classification the initial samples containing *Stentor* cells were lost. However, it looks very plausible that the ciliate specimens picked for our study were highly infected with these bacteria. The Stentor4 cell seemed to bear another peculiar endosymbiont, as numerous reads from the chloroplasts of the microalga *Mychonastes homosphaera* were detected in the dataset. This microalga species was documented in highly polluted wastewaters^65^, and was also found in symbiosis with another *Stentor* species, *S*. *polymorphus*^55^. Symbiotic *Chlorella*-like algae have been reported for *S*. *coeruleus* as well^66^. All selected *Stentor* cells contained blue-green pigment stentorin, specific only for *S*. *coeruleus*^67^, while presence or absence of symbiotic algae is not a reliable taxonomic character for the identification of *Stentor* species^68^. Some bacteria are known to have an algicidal effect, preventing algae survival and eventually killing them^69^, which could explain why the other six stentors heavily infected with “*Ca*. Megaira polyxenophila” did not contain microalgae. Still, it cannot be excluded that this particular *Stentor* cell belonged to another species. Anyway, removal of the chloroplast OTUs from the analysis revealed more uniform with other ciliates microbiome composition associated with Stentor4 cell.

Unexpectedly, few reads belonging to *Holospora obtusa* (*Proteobacteria*), bacterial intranuclear symbiont strictly host-specific for another ciliate, *Paramecium caudatum*, were detected in the dataset of the same *Stentor* cell (Supplementary Table 1). Outside of *Paramecium*, *Holospora* and related bacteria were reported in *Frontonia*^70,71^, but have never been recorded in *Stentor*^72^. *Holospora* possess an infectious stage in their life cycle, when they are released from the host cell to the environment and need to be ingested by another ciliate^72^. Probably, that *Stentor* cell might have engulfed *H*. *obtusa* infectious forms from the medium, and bacteria remained intact inside the ciliate up to DNA extraction. Of course, very low number of reads does not provide any basis to suggest that the symbiotic relationship emerged in this case. However, this finding gives interesting inkling to use 16S rRNA gene metabarcoding as molecular tool to investigate symbiont diversity in single cells of protists, and also to test early stages of symbiosis development when symbionts are just a few per host cell. On the other hand, the results of 16S rRNA gene metabarcoding analysis should be manually curated, and the best-matching sequences from the databases should be carefully checked, otherwise the presence of unknown symbiotic bacteria could be missed. Even known true symbionts might be misidentified, as it happened in our case with “*Ca*. Megaira polyxenophila”, which was misclassified initially with Ribosomal Database Project (RDP) against SILVA database as “uncultured *Rickettsiaceae*”.

After removal of reads of the most abundant symbionts, other OTUs became major or considerable enough to conclude that they reflect bacteria preferentially associated with ciliates. Many OTUs related to commensals and potential pathogens displayed high relative abundances within *Stentor* samples (Table 2). Bacteria discovered in all *Stentor* samples, according to the concept of core microbiome^53^, were supposed to form a core microbiome of *S*. *coeruleus*. *Capnocytophaga* sp., *Streptococcus* sp., some *Neisseriaceae* and *Porphyromonas* sp. were the most abundant, and, perhaps, they could be considered as keystone components of *S*. *coeruleus* core microbiome. These genera together with other members of *Stentor* core microbiome (e.g., *Veillonella sp*., *Rothia sp*., *Lautropia sp*., *Prevotella sp*., *Bergeyella* sp., and *Haemophilus* sp.) are a part of human microbiome^73^. Majority of them are known to colonize the mucosal surfaces of mammals, and are considered as opportunistic bacteria, if not true pathogens, which may cause serious diseases under certain conditions^56–60^. The interaction between *Streptococcus* sp. and *Veillonella* sp. was studied during the formation of biofilms^74^, which could be interesting since they have been both found in association with *Stentor* cells and might have a role in bacterial aggregation. The genus *Neisseria* and other representatives of the family *Neisseriaceae* were found in rather high abundancy (Table 2). *Neisseria* species normally colonize the mucosal surface of mammals and rarely invade their host cells^56,75^, so their presence in association with stentors might reveal novel aspects of this bacterial genus biology. Importantly, all these bacteria were absent or, like *Streptococcus sp*., present in much lower amounts in the environment.

Some free-living bacteria, like a representative of *Chitinophagaceae* or “Ca. Saccharibacteria” described in wastewater treatment plants^76^, were also retrieved in our analysis as a part of *Stentor* core microbiome (Table 2). Numerous OTUs associated with bacteria occasionally appearing as dominant microbiome components were defined in several *Stentor* samples. These bacteria belong either to presumably free-living genera (like bacterium MNG7 or *Xanthobacter* sp.), or to the groups of commensals (*Leptotrichiaceae*). We suggest to consider such bacteria as transitory components of *Stentor* microbiome, which by chance manage to colonize some ciliate cells.

We do not have an idea of any microbiome function useful for the host ciliate. Still, there is a common feature for almost all major bacteria in *Stentor* microbiome. These bacteria are very likely prone to interact with eukaryots, as they belong to genera encompassing numerous commensals or even pathogens of animals. When they appear freely in water, they may use their general skills to interact with available eukaryotic cells, namely, protists. There are two possible ways of bacterial persistence in association with protists: to stay attached on the surface of the host cell, or to resist digestion or even to escape from the food vacuoles to the host cytoplasm. Ciliates are rather big protists (*S*. *coeruleus* reaches up to 0.5–1 mm in length, Fig. 1), and their large cell surface, densely covered with cilia, may act as a kind of substrate for bacterial attachment, thus, facilitating them to propagate in a more stable environment. Bacteria propagating on ciliate cell surface have been described^77,78^. Sessile ciliates may colonize big areas of suitable substrates, and in this way bacterial community associated with ciliate cells may get stabilized in certain general environmental conditions. At the same time, ciliates can swim quickly and use different taxis allowing them to select favorable conditions^79^, which would be beneficial also for the associated bacteria. Ciliate surface may also offer a shelter to bacteria, protecting them from phagocytosis by other microorganisms. It is known that bacteria have developed numerous strategies to avoid prey-selective grazing by protozoa, namely morphological changes, high speed motility, and production of antagonistic or toxic substances^80–83^. One of the most important defense mechanisms is formation of biofilms or aggregations, in which separate bacteria are less vulnerable for predators^81^. From this point of view, the ciliate cell surface allows bacteria to adhere, aggregate, form biofilms and co-regulate their living activities through a quorum sensing mechanism, which regulates gene expression as a response to fluctuations in bacterial population density^84^. Thus, even a small substrate may be substantial for propagation of certain bacteria in the environment. To our best knowledge, no one have tried yet to analyze biofilms formation and bacterial quorum sensing mechanisms of regulation at such small-scale level as a surface of unicellular eukaryotes.

The second possibility for bacteria to stay in association with a ciliate is to somehow escape or survive digestion after being phagocytized. It is well known that many pathogenic bacteria enter the host cells by phagocytosis^85^. Some opportunistic bacteria, if engulfed by a phagocyte, are also able to avoid digestion. This may allow them to find a shelter from predators or from viral lysis inside the protozoan hosts, and to persist there as occasional “endosymbionts”^29^. For some bacteria, intracellular persistence may even trigger and increase their pathogenic properties and virulence^86^. Thus, protists may serve as transient reservoirs for many potentially dangerous bacteria. It has been proven that amoebae are the “Trojan horses” of the microbial world, as they act as temporary hosts for many pathogens^29^. At the same time, the similar role of ciliates has been also shown, but small-scale investigations were focused mainly on pathogens found as endosymbionts of ciliates^15,25^. In some recent works^49,61^ diverse digestion-resistant bacteria were described in different ciliates; some of them were enough numerous to be visualized inside the host cells with fluorescence *in situ* hybridization. However, the abundance of different bacteria forming the microbiome of ciliates is usually low, thus making NGS analysis a much more sensitive technique to study these phenomena.

Together with recently published data^41,42^ our results allow to presume that free-living ciliates may serve as certain “magnets” accumulating from the environment bacteria searching for hosts, and providing an ecological microniche for them. The ciliate core microbiomes then would be mostly formed by such bacteria, and the ratio between these would be dynamic and varying from cell to cell; sometimes also occasional bacteria could dominate, forming a transitory part of a microbiome. Actually this is what we obtained as a result of our metabarcoding analysis. Thus, ciliates can play in nature the role of reservoirs for potentially opportunistic and pathogenic bacteria.

Statistical analyses showed that sets of OTUs from the environmental controls were significantly different from those associated to stentors (Figs 5, 6). Indeed, statistical analyses, nmMDS and PCoA strongly confirmed that prokaryotic communities of *Stentor* cells and corresponding environments were clearly separated from each other, being very distant (Figs 5, 6). However, ciliate cells did not display a homogenous microbiome among themselves. When reads of abundant symbionts were removed from the analyses, it became clear that the microbiomes of single cells had some individual differences. At the same time, our data are insufficient to generalize if all specimens of the same ciliate species isolated from one locality possess similar or diverse microbiomes, but allow to suggest that there is a core microbiome for ciliates of the same species, at least isolated from the same locality. Further studies using several species of ciliates from the same location and from different origins should be performed to clarify this aspect. We still cannot rule out the hypothesis that environment determines the ciliates’ microbiomes, as all bacteria attracted and accumulated by ciliates, probably, come from their environment. However, we suppose that environment does not shape the core microbiomes of ciliates, as the latter in the end have almost nothing in common with the environmental prokaryotic community.

## Methods

### Sample collection and preparation

Three samples were collected from a sewage stream located in Peterhof, St. Petersburg, Russia (59.879780, 29.864358). The sampling points were located about 10 m downstream one after another, starting with PW1.

Each collected sample (200 ml) was divided in two subsamples: 150 ml were immediately filtered through 0.2 μm nitrocellulose filters (Sartorius, Germany) for further NGS analysis of the environmental controls, and 50 ml were brought to the laboratory within 15 min for isolation of ciliates. The filters were air-dried and stored at −80 °C till DNA extraction.

*Stentor* cells were observed *in vivo*, and identified as *S*. *coeruleus* (Fig. 1) by presence of blue-green pigment and moniliform macronucleus^67^. Three ciliate specimens were isolated as replicates for each sampling point with a sterile micropipette, washed thoroughly through several passages in sterile Volvic water and then kept in the last water aliquot overnight to reduce the load of random bacteria present in food vacuoles. Then stentors were washed briefly in autoclaved sterile distilled water, in order to reduce contaminants and microorganisms attached to the cell surface. Finally, single cells were fixed separately in 50 µl of 70% ethanol for further DNA extraction.

### DNA extraction, libraries preparation and sequencing

The filters were firstly treated by sonication as described^87^, then total genomic DNA was washed off and extracted using NucleoSpin® Tissue kit (Macherey-Nagel, Germany).

Just before DNA extraction, the *Stentor* samples were centrifuged for 30 min at 14000 rpm and 4 °C, and the pellet was dried for 3–5 minutes in the same PCR tube. Then 15 μl of MilliQ water and a mixture of glass beads (0.1 and 0.5 mm in diameter) were added to the tubes in approximate ratio 3:1. The mixture was homogenized with Tissue Lyser LT (QIAGEN, Germany) for 3 minutes at a maximal frequency of 50 Hz. The suspension was vortexed and centrifuged at 14000 rpm for 10 min. The lysate was transferred to a new tube, the glass beads were washed in 15 μl of MilliQ water, centrifuged again, and the supernatant was transferred in the tube with the lysate.

Preparation of the DNA libraries was performed according to the Illumina protocol (Part # 15044223, Rev. B.) using primers for V3 and V4 region of the 16S rRNA gene^88^. Two of nine *Stentor* samples did not yield sufficient DNA for library preparation and were discarded. The DNA libraries were sequenced on the MiSeq platform (Illumina, USA).

Preparation of the DNA libraries and sequencing was carried out in the Center of Shared Scientific Equipment “Persistence of microorganisms” at the Institute for Cellular and Intracellular Symbiosis, UrB RAS (Orenburg, Russia).

### Sequencing data analysis

Raw FASTQ files were analyzed using the Quantitative Insights Into Microbial Ecology 1.9.1 software package (QIIME)^89^. De-multiplexing and quality filtering were performed removing any low quality or ambiguous reads, and sequences shorter than 200 nucleotides were discarded. Chimeras were identified using UCHIME^90^ and subsequently removed from the analysis. Operational Taxonomic Units (OTUs) were clustered with a 97% of similarity cutoff using USEARCH^91^, and SILVA 119 database^92^ as reference. The most common sequence was selected in each OTU as representative. Taxonomic classification up to genus level was performed using Ribosomal Database Project Classifier (RDP)^93^ against the SILVA 119 database.

### Prokaryotic community analysis

QIIME was used to evaluate diversity of the prokaryotic communities both within and between samples. Firstly, the relative abundances of prokaryotic taxa were estimated as the percentage of contigs number for each taxon for the environmental and the *Stentor* cell samples, and the community richness was evaluated calculating the Shannon index. The significance of OTUs presence in the ciliates samples was assessed using the non-parametric Kruskal-Wallis test. Furthermore, similarities between stentors and their environments were assessed using the non-metric Multi-Dimensional Scaling (nmMDS) and Principal Coordinate Analysis (PCoA) based on the unweighted Unifrac distance matrices. In addition, beta diversity was estimated to compare the bacterial communities of the *Stentor* cells and their environments. To confirm the significance of differences observed between bacterial communities of the environments and stentors, ANOSIM was calculated. The same analyses were carried out also after excluding the most abundant OTUs representing symbiotic microorganisms associated with stentors (see Results).

## Supplementary information

Dataset 1

## Data Availability

The raw reads generated and analyzed during the current study are available in the ENA database (study number PRJEB30974). The datasets analyzed during this study are included in the Supplementary Information file.
